# Recognizing the Top Peer Reviewers in 2025 for the *Journal of Virology*

**DOI:** 10.1128/jvi.01010-26

**Published:** 2026-08-18

**Authors:** Felicia Goodrum, Stacey Schultz-Cherry

**Affiliations:** 1 Geisel School of Medicine, Dartmouth College 12285 , Hanover, New Hampshire, USA; 2 Infectious Diseases, St. Jude Children’s Research Hospital 5417, Memphis, Tennessee, USA

## EDITORIAL

The Journal of Virology (JVI) remains the leading journal in virology, with 77,299 citations in 2025, reaffirming its status as the most cited journal in the Virology category, according to the latest *Journal Citation Reports* (Clarivate, 2025). This excellence is derived directly from JVI authors, reviewers, editors, and staff. The editors, editorial board members, and reviewers are to be commended for their hard work in ensuring a smooth review process that is constructive, rigorous, fair, and objective, as well as expedient. The median time from manuscript submission to an editor’s ﬁrst peer review decision letter is 31 days.

We thank all the JVI reviewers over the past year. We would especially like to thank the following 42 reviewers, whose service to JVI during the past year was exceptional in the total number of manuscripts reviewed and in the expediency of submitting their reviews on time, with an average of 11 days or fewer. All of these reviewers completed 10 or more reviews in 2025. A special shout out goes to the reviewers who were also on the 2024 Top Reviewer list (individuals listed in bold).

**Matthew Aliota, University of Minnesota Twin Cities**

Susan C. Baker, Loyola University Chicago

Bruce W. Banfield, Queen’s University

**David J. Barton, University of Colorado Anschutz School of Medicine**

Scott B. Biering, University of California San Diego

Karl W. Boehme, University of Arkansas for Medical Sciences

Adrianus C. Boon, Washington University in St. Louis School of Medicine

Heather M. Callaway, Montana State University

**Nora M. Chapman, University of Nebraska Medical Center College of Medicine**

Mingzhou Chen, Hubei University

**Xufang Deng, Oklahoma State University**

Qiang Ding, Tsinghua University

Oystein Evensen, Norwegian University of Life Sciences

Benjamin Gewurz, Harvard Medical School, Brigham and Women’s Hospital

**Douglas Paul Gladue, Seek Labs**

Haitao Guo, University of Pittsburgh

**Ju-Tao Guo, Baruch S. Blumberg Institute**

Peter Halfmann, University of Wisconsin-Madison

Jianming Hu, Penn State College of Medicine

**Ronald M. Iorio, University of Massachusetts Medical School**

**Dong-Yan Jin, The University of Hong Kong**

**Florian Krammer, Icahn School of Medicine at Mount Sinai**

Bin Li, Jiangsu Academy of Agricultural Sciences Institute of Veterinary Medicine

Feng Li, University of Kentucky

Xiang-Jin Meng, Virginia Polytechnic Institute and State University

Joe S. Mymryk, University of Western Ontario

Robert C. Orchard, The University of Texas Southwestern Medical Center

**Richard K. Plemper, Georgia State University**

Alexander Ploss, Princeton University

**Stefan H. Pöhlmann, Deutsches Primatenzentrum GmbH - Leibniz-Institut fur Primatenforschung**

John G. Purdy, The University of Arizona

**Raymond R. Rowland, Kansas State University**

Tongling Shan, Shanghai Veterinary Research Institute, Chinese Academy of Agricultural Sciences

**Kenneth A. Stapleford, New York University Grossman School of Medicine**

Anastasia N. Vlasova, The Ohio State University

**Penghua Wang, University of Connecticut School of Medicine**

Friedemann Weber, Justus-Liebig University Gießen

Lok-Yin Roy Wong, Rutgers Biomedical and Health Sciences

**Xianfang Wu, Cleveland Clinic**

**Shuqi Xiao, Lanzhou Veterinary Research Institute**

Xuping Xie, The University of Texas Medical Branch at Galveston

Sonia Zuñiga, Centro Nacional de Biotecnologia

